# Comparing clinical outcomes of ARB and ACEi in patients hospitalized for acute COVID-19

**DOI:** 10.1038/s41598-023-38838-8

**Published:** 2023-07-21

**Authors:** Seiji Hamada, Tomoharu Suzuki, Yasuharu Tokuda, Kiyosu Taniguchi, Kenji Shibuya

**Affiliations:** 1 Urasoe General Hospital, Urasoe, Okinawa Japan; 2 The Tokyo Foundation for Policy Research, Minato-ku, Tokyo, Japan; 3 Muribushi Okinawa Center for Teaching Hospitals, 3-42-8 Iso, Urasoe, Okinawa 901-2132 Japan; 4 National Hospital Organization Mie National Hospital, Tsu, Mie Japan; 5 University of Tsukuba School of Medicine, Tsukuba, Japan

**Keywords:** Epidemiology, Outcomes research

## Abstract

Continued receipt of Renin–Angiotensin–Aldosterone inhibitors in patients with COVID-19 has shown potential in producing better clinical outcomes. However, superiority between ACEi (angiotensin-converting enzyme inhibitors) and ARB (angiotensin II receptor blockers) regarding clinical outcomes in this setting remains unknown. We retrospectively collected data on patients hospitalized for acute COVID-19 using the nationwide administrative database (Diagnosis and Procedure Combinations, DPC). The DPC data covered around 25% of all acute care hospitals in Japan. Patient outcomes, with focus on inpatient mortality, were compared between patients previously prescribed ACEi and those prescribed ARB. Comparisons based on crude, multivariate and propensity-score adjusted analysis were conducted. We examined a total of 7613 patients (ARB group, 6903; ACEi group 710). The ARB group showed lower crude in-hospital mortality, compared to the ACEi group (5% vs 8%; odds ratio, 0.65; 95% CI 0.48–0.87), however not in the multivariate-adjusted model (odds ratio, 0.95; 95% CI 0.69–1.3) or propensity-score adjusted models (odds ratio, 0.86; 95% CI 0.63–1.2). ARB shows potential in reducing hospital stay duration over ACEi in patients admitted for COVID-19, but does not significantly reduce in-hospital mortality. Further prospective studies are needed to draw a definitive conclusion, but continuation of either of these medications is warranted to improve clinical outcomes.

## Introduction

As of 25 April 2022, SARS-CoV-2, the novel coronavirus responsible for the COVID-19 pandemic, has officially claimed 6.2 million lives and continues to pose public health risk on a global scale^1^. Further, as of May 2022, the World Health Organization estimated that global excess deaths associated with the pandemic between Jan 2020 and Dec 2021 were 14.91 million^2^. SARS-CoV-2 gains entry into human cells by binding to the receptor ACE2, part of the counter-regulating axis of the Renin–Angiotensin–Aldosterone system (RAAS)^3,4^. While decreasing ACE2 expression would prohibit virus entry and thereby reduce the risk of infection, it would also decrease favorable hemostatic effects of ACE2 leading to exacerbation of disease severity^5^. The use of ACEIs (angiotensin-converting enzyme inhibitors) and ARBs (angiotensin II receptor blockers) have been shown in animal models to increase ACE2 expression^6,7^, thus leading to controversy over its continued use in patients with COVID-19. A few studies have suggested an increased risk of severe disease^8,9^, while a recent large-scale randomized controlled trial comparing controlled placebo and ARB, displayed no clear benefit on clinical outcomes^10^. However, the majority of observational studies showed no association of increased risk of severe disease, and some have even displayed beneficial clinical outcomes^11,12^. In a systematic review and meta-analysis consisting of 101,949 COVID-19 patients, 26,545 of whom were receiving ACEIs or ARBs, a significant reduction in the risk of death (adjusted OR [aOR], 0.57; 95% CI 0.43–0.76; *p* < 0.001) and severe adverse events (aOR, 0.68; 95% CI 0.53–0.88; *p* < 0.001) was observed after adjustment for covariates, hinting at potential clinical benefit^13^.

Therefore, although a definitive conclusion is yet to be made, the current narrative is that the use of Renin–Angiotensin–Aldosterone inhibitors exerts potential clinical benefit in COVID-19 patients. Provided both improve mortality, either could be used more favorably at the time of developing COVID-19, however, clinical superiority between the two in this setting remains to be elucidated. We retrospectively analyzed data of patients admitted for acute COVID-19 who were either on ACEI or ARB prescription and compared clinical outcomes.

## Methods

### Study population

The current study used the largest nationwide hospital administrative database, called diagnosis and procedure combination (DPC) data in Japan, from 25% (438 of 1750) of all acute care hospitals. The majority of middle to large-sized hospitals are part of the DPC register. The DPC dataset used in the current study was collected from the Ministry of Health, Welfare, and Labour of the government of Japan by the Medical Data Vision (MDV), Co., Tokyo, Japan (As of April 1, 2021) ^14^. The MDV DPC dataset covers DPC hospitals throughout Japan, and the age and gender distribution of registered patients are comparable to the distribution of patients at healthcare institutions nationwide as published by the government of Japan, thus is a fair representation of the national data^14^. Recent studies on COVID-19 used data of an MDV DPC dataset^15,16^. The current study collected the MDV DPC data from 1 January 2010 to 30 November 2021 and included patients of all ages with confirmed (RT-PCR test positive) acute COVID-19 (International Classification of Diseases and Related Health Problems, 10th Revision diagnosis code U071) who required admission with acute COVID-19 as the major cause of admission. Data from the first such admission was used in the current study.

### Data collection

The MDV DPC dataset for our analysis included patients’ demographics (age and gender), diagnoses, comorbidities, prescriptions, and procedures as well as clinical data including outcomes (e.g., in-hospital mortality) and length of hospital stay. Patients on ACEi or ARB were identified as those who were prescribed these medications within 30 days prior to the date of admission related to acute COVID-19. Patients on neither ACEi nor ARB were excluded. Patients on both ACEi and ARB were excluded from analysis because this combination is not recommended in any clinical scenario. Our data included demographics, smoking history, body mass index, baseline disease, and confirmed presence of critical conditions at the time of admission by physicians (shock, coma, or cardiorespiratory failure). Baseline disease data were identified and collected using ICD-10 coding based on the previous study that used MDV DPC data^15^. Since there was data missing for smoking history and body mass index, multiple imputations with chained equations were used to utilize data of all available admitted patients^15^. Our data also included complications (acute respiratory distress syndrome [ARDS], etc.), treatment modalities, requirement for oxygen therapy, invasive mechanical ventilation or renal replacement therapy (RRT), as well as in-hospital mortality and length of hospital stay. In the DPC database, information on prescribing physicians’ characteristics for ACEi or ARB (specialty, age, gender) was unavailable. The current study was approved by the Muribushi Okinawa Ethics Committee.

### Statistical analyses

Regarding baseline characteristics, continuous variables were described as mean and standard deviation, while nominal variables as count and proportion. Logistic or linear regression models were employed to examine the association between outcomes (in-hospital mortality and requirement of invasive mechanical ventilation, or renal replacement therapy, and length of hospital stay) and the use of ACEi or ARB. The models were constructed by crude comparison, adjusted analysis for baseline clinical characteristics (including age, gender, obesity, referral from another institution, ischemic heart disease, heart failure, arrythmia, hypertension, dementia, dyslipidemia and iron deficiency anemia), and propensity score analyses (score adjusted). The propensity score analysis was conducted based on the method of previous studies^17,18^. On analysis of RRT, chronic kidney disease was omitted from variables for adjustment because of interaction to ARB. Patients with hemodialysis before admission (n = 108; ARB = 106, ACEi = 2) was excluded for analysis in RRT. Analyses were conducted using STATA version 15 (NC, US). A *p*-value less than 0.05 was defined as statistically significant.

## Results

### Baseline characteristics

Between January 2020 and November 2021, 67,348 inpatients were diagnosed with COVID-19, 7613 patients of which were receiving either ARB or ACEi (ARB group = 6193, ACEi group = 710) and were included in the study. Compared to patients on ACEi, those on ARB were more likely to be younger, women, obese, hypertensive, or suffering from chronic kidney disease (CKD grade 5D). Meanwhile, the ACEi group was more likely to have a history of ischemic heart disease, heart failure, arrhythmia, dementia, or iron deficiency anemia (Table 1). There was no statistically significant difference in smoking history, history of diabetes mellitus, cerebrovascular disease, chronic kidney disease (except CKD grade 5D), peripheral vascular disease, liver cirrhosis or cancer.

**Table 1:** Baseline characteristics of participants at admission in Japan for COVID-19.

	ARB (N = 6903)	ACEi (N = 710)	*p*-value
Age	70 (58–80)	73 (61–84)	< 0.001
Range of age (years-old)			< 0.001
0–10	0 (0%)	2 (0.28%)	
10–20	2 (0.029%)	4 (0.56%)	
20–30	22 (0.32%)	5 (0.70%)	
30–40	121 (1.8%)	12 (1.7%)	
40–50	600 (8.7%)	36 (5.1%)	
50–60	1317 (19%)	113 (16%)	
60–70	1473 (21%)	131 (18%)	
70–80	1823 (26%)	172 (24%)	
80–90	1244 (18%)	176 (25%)	
90–100	300 (4.3%)	59 (8.3%)	
Male sex	4257 (62%)	466 (66%)	0.038
Smoking history	3162 (46%)	326 (46%)	0.96
Obesity (≥ 30)	1069 (15%)	74 (10%)	< 0.001
Referral from another institution	596 (8.6%)	77 (11%)	0.048
Comorbidities or past medical history			
Ischemic heart disease	809 (12%)	169 (24%)	< 0.001
Diabetes mellitus	3051 (44%)	326 (46%)	0.38
Cerebrovascular disease	413 (6.0%)	43 (6.1%)	0.94
Heart failure	953 (14%)	255 (36%)	< 0.001
Arrhythmia	518 (7.5%)	126 (18%)	< 0.001
Chronic kidney disease	607 (8.8%)	60 (8.5%)	0.76
CKD grade 1	1 (0.014%)	0 (0%)	0.75
CKD grade 2	5 (0.072%)	1 (0.14%)	0.54
CKD grade 3	4 (0.058%)	1 (0.14%)	0.41
CKD grade 3a	14 (0.20%)	2 (0.28%)	0.66
CKD grade 3b	26 (0.38%)	2 (0.28%)	0.69
CKD grade 3 overall	44 (0.64%)	5 (0.70%)	0.83
CKD grade 4	33 (0.48%)	2 (0.28%)	0.46
End-stage CKD	145 (2.1%)	11 (1.5%)	0.32
CKD grade 5	15 (0.22%)	1 (0.14%)	0.67
CKD grade 5D	46 (0.67%)	0 (0%)	0.029
CKD grade 5 overall	206 (3.0%)	12 (1.7%)	0.049
Hemodialysis prior to admission	71 (1.0%)	2 (0.28%)	0.052
Dementia	457 (6.6%)	87 (12%)	< 0.001
Dyslipidemia	2567 (37%)	295 (42%)	0.022
Iron deficiency anemia	616 (8.9%)	91 (13%)	< 0.001
Peripheral vascular disease	79 (1.1%)	10 (1.4%)	0.53
Liver cirrhosis	47 (0.68%)	3 (0.42%)	0.42
Cancer	391 (5.7%)	49 (6.9%)	0.18

Data are presented as median (IQR) for continuous measures, and n (%) for categorical measures. Mann–Whitney U test was used for comparing nonparametric variables, Student's t-test was used for comparing continuous variables, and chi-square test or Wilcoxon rank-sum test for comparing categorical variables. *ACEi* angiotensin-converting enzyme inhibitor, *ARB* angiotensin II receptor blocker. Missing data: Age = 1 (ARB = 1, ACEi = 0).

### The difference in treatment and complication of hospitalized patients with COVID-19 between ARB and ACEi

As shown in Table 2, the ARB group was more likely to receive dexamethasone or other glucocorticoids and renal replacement therapy (RRT, including patients on hemodialysis before admission) while less likely to receive invasive mechanical ventilation (IMV), extracorporeal membrane oxygenation, vasopressors or blood transfusion. There was no statistically significant difference in the proportion of oxygen therapy, non-invasive positive pressure therapy or renal replacement therapy in patients without hemodialysis before admission (165/6797 [2.4%] vs 13/708 [1.8%]). In terms of the short-term complication of COVID-19 (in 30 days after the diagnosis of COVID-19), patients on ARB were less likely to experience deep vein thrombosis (DVT) and acute ischemic heart disease (aIHD) or atrial fibrillation/flutter. While no difference was observed in septic shock, acute kidney injury (AKI), disseminated intravascular coagulation (DIC), pulmonary embolism (PE), cardiopulmonary arrest (CPA), acute myocarditis, subarachnoid/intracranial hemorrhage (SAH/ICH) or brain infarction (BI). As long-term complications (30 days or more after COVID-19 diagnosis), ARB groups experienced less DVT, and no statistically significant difference was observed in other complications (septic shock, AKI, PE, CPA, aIHD, SAH/ICH or BI). The proportion of acute respiratory distress syndrome (ARDS) after admission was lower in the ARB group.

**Table 2:** Treatment, complications, and clinical outcome of hospitalized patients in Japan for COVID-19.

	ARB (N = 6903)	ACEi (N = 710)	*p*-value
Medications for treatment			
Dexamethasone or other steroids	2760 (40%)	245 (35%)	0.004
Tocilizumab	232 (3.4%)	22 (3.1%)	0.71
Dexamethasone and tocilizumab	103 (1.5%)	12 (1.7%)	0.68
Remdesivir	8 (0.12%)	0 (0%)	0.36
Ivermectin	15 (0.22%)	1 (0.14%)	0.67
Supportive medical care			
Oxygen therapy (overall)	3912 (57%)	395 (56%)	0.60
Oxygen therapy with nasal cannula or mask	3872 (56%)	384 (54%)	0.31
NPPV	15 (0.22%)	1 (0.14%)	0.21
Invasive mechanical ventilation	343 (5.0%)	58 (8.2%)	< 0.001
ECMO	12 (0.17%)	5 (0.70%)	0.004
Vasopressor	206 (3.0%)	56 (7.9%)	< 0.001
Renal replacement therapy (RRT)	258 (3.7%)	15 (2.1%)	0.027
Blood transfusion	112 (1.6%)	33 (4.6%)	< 0.001
ICU admission	524 (7.6%)	63 (8.9%)	0.22
Medical conditions on admission			
Coma on admission	21 (0.30%)	2 (0.28%)	0.92
Shock on admission	2 (0.03%)	0 (0%)	0.65
Respiratory or circulatory failure on admission	3482 (50%)	362 (51%)	0.78
Metabolic derangement on admission	23 (0.33%)	1 (0.14%)	0.38
Complications in 30 days after admission/diagnosis of COVID-19 (data available to Nov 2021)			
ARDS in 30 days after admission	160 (2.3%)	27 (3.8%)	0.015
Septic shock in 30 days after COVID-19 diagnosis	38 (0.55%)	4 (0.56%)	0.96
Acute kidney injury in 30 days after COVID-19 diagnosis	63 (0.91%)	10 (1.4%)	0.20
Disseminated intravascular coagulation in 30 days after COVID-19 diagnosis	224 (3.2%)	29 (4.1%)	0.23
Deep vein thrombosis in 30 days after COVID-19 diagnosis	1023 (15%)	128 (18%)	0.023
Pulmonary embolism in 30 days after COVID-19 diagnosis	217 (3.1%)	26 (3.7%)	0.45
Cardiopulmonary arrest in 30 days after COVID-19 diagnosis	13 (0.19%)	1 (0.14%)	0.78
Acute ischemic heart disease in 30 days after COVID-19 diagnosis	70 (1.0%)	27 (3.8%)	< 0.001
Acute myocarditis in 30 days after COVID-19 diagnosis	2 (0.03%)	1 (0.14%)	0.15
Atrial fibrillation/flutter in 30 days after COVID-19 diagnosis	477 (6.9%)	98 (14%)	< 0.001
SAH or ICH in 30 days after COVID-19 diagnosis	27 (0.39%)	2 (0.28%)	0.65
Brain infarction in 30 days after COVID-19 diagnosis	218 (3.2%)	19 (2.7%)	0.48
Patient outcome			
Length of stay	11 (8–17)	13 (9–20)	< 0.001
In-hospital mortality	356 (5.2%)	55 (7.7%)	0.004
Tracheostomy	36 (0.5%)	10 (1.4%)	0.004

Data are presented as median (IQR) for continuous measures, and n (%) for categorical measures. Pearson's chi-square test was employed for comparing binary variables, while Mann–Whitney U test was used for comparing nonparametric continuous variables.

Oxygen therapy (overall) includes oxygen therapy with nasal cannula or mask, non-invasive positive pressure therapy, MV and ECMO.

*ACEi* angiotensin-converting enzyme inhibitor, *ARB* angiotensin II receptor blocker, *ARDS* acute respiratory distress syndrome, *ECMO* extracorporeal membrane oxygenation, *ICH* intracranial hemorrhage, *ICU* intensive care unit, *NPPV* non-invasive positive pressure ventilation, *SAH* subarachnoid hemorrhage.

Patients on maintenance hemodialysis before admission (n = 108; ARB = 106, ACEi = 2) was excluded for the analysis about renal replacement therapy.

### The difference in the clinical outcome of hospitalized patients with COVID-19 between ARB and ACEi

The patients who received ARB showed lower crude in-hospital mortality (356/6903 [5%] vs 55/710 [8%]), but multiple logistic regression analyses and analysis based on propensity score showed no difference in in-hospital mortality between the ARB and ACEi groups (Table 3). The ARB group showed lower crude odds of ARDS but not in adjusted models. In the ARB group, the odds of aIHD was lower in crude and propensity score analysis. Prevalence of IMV in crude, multivariate and propensity score analysis by baseline characteristics such as past medical history was lower in ARB patients (Crude: 343/6903 [5%] vs 58/710 [8%], OR 0.59 [95% CI 0.44–0.79], Multivariate adjusted: OR 0.67 [0.49–0.91], Propensity score-adjusted: OR 0.68 [0.50–0.92]). Regarding RRT, although there was no difference in unadjusted prevalence between ARB and ACEi, multivariate and propensity score-adjusted analysis, after omitting patients who were already on hemodialysis before admission, revealed that RRT was more likely to be employed in the ARB group (Multivariate adjusted: OR 2.1 [95% CI 1.2–3.9], Propensity score-adjusted: OR 2.4 [95% CI 1.1–3.7]). The ARB group showed a shorter median length of hospital stay (11 days [IQR 8–17] vs 13 days [IQR 9–20], Coefficient: − 3.8 days [− 4.8 to − 2.8], Tables 2, 3), which was still significant after multivariate or propensity score adjustment. (Table 3).

**Table 3:** Comparison of unadjusted, adjusted, fully adjusted and propensity score adjusted clinical outcomes between patients with ARB compared to ACEi.

Outcome	Unadjusted			Multivariable adjusted			Propensity score adjusted		
	OR/coefficient	95% CI	*p*-value	OR/coefficient	95% CI	*p*-value	OR/coefficient	95% CI	*p*-value
Invasive mechanical ventilation	0.59	0.44–0.79	< 0.001	0.67	0.49–0.91	0.011	0.68	0.50–0.92	0.013
Renal replacement therapy	1.3	0.75–2.35	0.33	2.1	1.2–3.9	0.012	2.4	1.1–3.7	0.018
Acute respiratory distress syndrome	0.60	0.40–0.91	0.016	0.76	0.49–1.2	0.23	0.75	0.49–1.2	0.20
Deep vein thrombosis	0.79	0.65–0.97	0.023	0.84	0.68–1.0	0.68	0.85	0.69–1.04	0.12
Acute ischemic heart disease	0.26	0.17–0.41	< 0.001	0.60	0.36–1.0	0.052	0.53	0.32–0.88	0.014
Atrial fibrillation/flutter	0.46	0.37–0.58	< 0.001	0.91	0.63–1.3	0.59	0.88	0.68–1.1	0.36
In-hospital mortality	0.65	0.48–0.87	0.004	0.95	0.69–1.3	0.77	0.86	0.63–1.2	0.35
Length of hospital stay	− 3.8	− 4.8–− 2.8	< 0.001	− 2.8	− 3.8–− 1.8	< 0.001	− 2.8	− 3.8–− 1.8	< 0.001

*OR* odds ratio, *RRT* renal replacement therapy. Logistic regression was implemented for analysis of mechanical ventilation and in-hospital mortality, and linear regression was employed for comparing length of hospital stay. "Multivariate adjusted" analysis was adjusted for age, male sex, obesity (Body mass index over 30), referral from another healthcare institution and comorbidities (hypertension, chronic kidney disease, ischemic heart disease, heart failure, arrhythmia, dementia, dyslipidemia and iron deficiency anemia). Propensity score was calculated from following variables: age, male sex, obesity (Body mass index over 30), referral from another healthcare institution and comorbidities (hypertension, ischemic heart disease, heart failure, arrhythmia, dementia, dyslipidemia and iron deficiency anemia). In analysis of RRT, chronic kidney disease was omitted from variables for adjustment because of interaction to ARB. Patients with hemodialysis before admission (n = 108; ARB = 106, ACEi = 2) was excluded for analysis in RRT.

## Discussion

To the best of our knowledge, our study comprising of 7613 patients with acute COVID-19, is the first large-scale study of its kind to directly compare clinical outcomes between ACEi and ARB, while other available clinical studies are limited to hypotheses based on indirect comparisons. Our results showed that in crude analysis, those receiving ARB had a lower rate of in-hospital mortality, however, no superiority between the two was seen after adjustments for age, gender, obesity, referral from another institution, ischemic heart disease, heart failure, arrythmia, hypertension, dementia, dyslipidemia, and iron deficiency anemia. Notably, in secondary analysis, significant reduction in hospital stay duration was seen in the ARB group in both adjusted and unadjusted analysis. This may be partly explained by the fact the ARB group was associated with reduced risk of multiple clinical outcomes, with unadjusted risk reduction in deep vein thrombosis and atrial arrhythmia observed, while unadjusted and adjusted risk reduction seen for acute ischemic heart disease and invasive mechanical ventilation, without statistically significant difference for acute respiratory distress syndrome. A recent study suggested that ACEIs were associated with increased odds of sepsis in COVID-19 patients, while ARBs were not^19^. In addition to vasodilation, ARBs mediate anti-inflammatory effects^20^, which may contribute to better outcomes among COVID-19 patients. In a meta-analysis of 7 randomized controlled trials, the use of ACEi/ARB in COVID-19 patients was not associated with a higher risk of mortality (risk ratio [RR] = 0.84, 95% CI 0.57–1.22, *p* = 0.10, I2 = 43%). Moreover, in the same study, compared with no ACEi/ARB use, ARBs were associated with a significant decrease in mortality ([RR] = 0.23, 95% CI 0.09–0.60, *p* = 0.55, I2 = 0%) and disease severity ([RR] = 0.38, 95% CI 0.19–0.77, *p* = 0.007), hinting indirectly at potential superiority to ACEi^21^. In another recent prospective study that included 566 hypertensive patients with acute COVID-19, reduced risk of in-hospital mortality in those receiving ARB to those receiving other blood pressure lowering agents was demonstrated, further hinting indirectly at potential superiority to ACEi^22^. Although a definitive conclusion cannot be drawn, our findings are in line with these studies, pointing further at possible benefit of ARBs in this clinical setting.

Significant differences in baseline characteristics between the two groups were seen, most notably, those on ACEi were more likely to be suffering from cardiac complications including ischemic heart disease, heart failure and arrhythmia. Multiple professional cardiology societies (including the American Heart Association, American College of Cardiology, European Society of Cardiology) recommend the use of ACEi to reduce the risk of developing symptomatic heart failure and mortality in those with LVEF ≤ 40%, with ARBs reserved for those who are intolerant to ACEi. This is suggestive of the fact that those on ACEi are more likely to already suffer from cardiac disease, thereby relatively decreasing the risk of developing acute ischemic heart disease (aIHD) in the ARB group, as is shown in our results. Patients on NPPV were rarely seen in both groups, likely due to the national Japanese guideline on COVID-19 published during this period recommending against the use of NPPV to address the possible risk of aerosols being contaminated with SARS-CoV2 particles.

There are several limitations to our study. First, our study is of retrospective nature, and a further prospective study that directly compares ACEi and ARB is needed for a more definitive conclusion. Second, the baseline population was considerably greater in the ARB group which may have led to selection bias and outcomes may be disproportionate despite propensity-matched analysis. Although propensity-score matching and multivariable regression models were utilized in the statistical analysis to account for measured confounders, due to the study's observational nature, some confounders may exist that may not have been accounted for. As mentioned above, the ACEi population were more likely to be suffering from multiple comorbidities especially heart disease which may hint towards a sicker baseline population, yet we were unable to match propensity scores completely which may have resulted in potentially biased results. Third, we were unable to extract data on antibiotic prescriptions between the two groups which may introduce a source of potential confounding, therefore, results should be interpreted with caution. Fourth, our DPC data does not cover the entire Japanese population thus our results can only be interpreted as a substitute representation, therefore, a selection bias may exist within the study population. It also remains to be seen whether the results are representative of smaller, rural populations. Lastly, information regarding vaccine status was not included in the analysis, which may have had an impact on clinical outcomes.

In conclusion, among hypertensive patients hospitalized for COVID-19, ARB was associated with lower crude rate of in-hospital mortality, but not in adjusted analysis. The use of ARB has potential in improving clinical outcomes, but continuation of either ARB or ACEi is of utmost importance.

## Data Availability

The data that support the findings of this study are available from the Medical Data Vision, Co., but restrictions apply to the availability of these data, which were used under license for the current study, and so are not publicly available. Data are however available from the authors upon reasonable request and with permission of Medical Data Vision, Co.
